# HLA Variation and SARS-CoV-2 Specific Antibody Response

**DOI:** 10.3390/v15040906

**Published:** 2023-03-31

**Authors:** Dawit Wolday, Chun Yiu Jordan Fung, Gregory Morgan, Selina Casalino, Erika Frangione, Jennifer Taher, Jordan P. Lerner-Ellis

**Affiliations:** 1 Pathology and Laboratory Medicine, Mount Sinai Hospital, Sinai Health, Toronto, ON M5G 1Z5, Canada; jordan.fung@sinaihealth.ca (C.Y.J.F.); greg.morgan@sinaihealth.ca (G.M.); selina.casalino@sinaihealth.ca (S.C.); erika.frangione@sinaihealth.ca (E.F.); jennifer.taher@sinaihealth.ca (J.T.); 2 Lunenfeld-Tanenbaum Research Institute, Sinai Health, Toronto, ON M5G 1Z5, Canada; 3 Department of Laboratory Medicine and Pathobiology, University of Toronto, Toronto, ON M5G 1Z5, Canada

**Keywords:** COVID-19, SARS-CoV-2, antibody, immunoglobulin, HLA, MHC, polymorphism, genomics

## Abstract

Differences in SARS-CoV-2-specific immune responses have been observed between individuals following natural infection or vaccination. In addition to already known factors, such as age, sex, COVID-19 severity, comorbidity, vaccination status, hybrid immunity, and duration of infection, inter-individual variations in SARS-CoV-2 immune responses may, in part, be explained by structural differences brought about by genetic variation in the human leukocyte antigen (HLA) molecules responsible for the presentation of SARS-CoV-2 antigens to T effector cells. While dendritic cells present peptides with HLA class I molecules to CD8+ T cells to induce cytotoxic T lymphocyte responses (CTLs), they present peptides with HLA class II molecules to T follicular helper cells to induce B cell differentiation followed by memory B cell and plasma cell maturation. Plasma cells then produce SARS-CoV-2-specific antibodies. Here, we review published data linking HLA genetic variation or polymorphisms with differences in SARS-CoV-2-specific antibody responses. While there is evidence that heterogeneity in antibody response might be related to HLA variation, there are conflicting findings due in part to differences in study designs. We provide insight into why more research is needed in this area. Elucidating the genetic basis of variability in the SARS-CoV-2 immune response will help to optimize diagnostic tools and lead to the development of new vaccines and therapeutics against SARS-CoV-2 and other infectious diseases.

## 1. Introduction

SARS-CoV-2 infection leads to a diverse spectrum of clinical outcomes ranging from asymptomatic to critical clinical presentation and death. Established factors influencing these diverse clinical outcomes include age, sex, racial ancestry, comorbidities, coinfections, and SARS-CoV-2 variants [1,2,3,4,5,6,7]. Additionally, genetic factors may play a significant role in the pathogenesis of COVID-19-associated severity, reviewed in [8].

The human leukocyte antigen (HLA) system plays a crucial role in the host’s immune response during an encounter with an infectious agent [9]. The HLA system is further classified into HLA class I (HLA-A, HLA-B, and HLA-C) and HLA class II (HLA-DR, HLA-DQ, and HLA-DP). Whereas HLA class I is involved in the presentation of antigens in an endogenous pathway, HLA class II is engaged in an exogenous antigen presentation pathway [9]. HLA class I molecules are expressed on all nucleated cells and HLA class II molecules are expressed on specialized or professional antigen-presenting cells (APCs), such as dendritic cells, macrophages, and mature B cells. The HLA genes are located within the major histocompatibility complex (MHC). In humans, the MHC gene locus located in the short arm of chromosome six is among the most complex systems in humans. There are more than 15,000 genetic variations in the HLA class I and class II genes [9]; this, combined with the heterogeneity in antibody response and the variability of analytic methods have made it difficult to study. Nevertheless, among the genetic factors that determine the clinical outcome upon exposure to antigens, the HLA system plays a pivotal role in the immune response to various infections, such as viral hepatitis, dengue, HIV-1, *Mycobacterium tuberculosis*, and malaria, as well as small pox, rotavirus, measles-mumps-rubella, and influenza vaccines [10,11,12,13,14,15,16,17,18,19,20,21,22]. Likewise, emerging data show that polymorphisms in the HLA system may confer protection from or susceptibility to infection and severe disease in patients with SARS-CoV-2 infection, reviewed in [23,24]. In addition, the genetic makeup of an individual host may also modulate the response to SARS-CoV-2 vaccination [24,25]. Indeed, differences in SARS-CoV-2-specific immune responses have been observed between individuals following SARS-CoV-2 infection or vaccination [26,27,28,29], and HLA variation may contribute to such differences [24,25].

Understanding the underlying mechanisms of the relationship between HLA variation and SARS-CoV-2-specific antibody responses may provide an avenue for the development of novel therapeutic and preventive strategies for SARS-CoV-2, and consequently, the prevention of long-term sequelae of SARS-CoV-2 infection. In this article, we review the current evidence and discuss future prospects.

## 2. Host Immune Response to SARS-CoV-2: Innate and Adaptive Immunity

SARS-CoV-2 enters the respiratory tract’s airway epithelial cells via the angiotensin-converting enzyme 2 (ACE2)—the host receptor for the receptor-binding domain (RBD) of the spike protein of SARS-CoV-2 [30]. The cell surface-associated transmembrane serine protease (TMPRSS2) regulates the binding of the RBD to the ACE2 receptor that eventually triggers endocytosis of the virus, followed by the release of the viral mRNA into the host cells’ cytoplasm [31]. Within the cytoplasm, the virus hijacks the host cell machinery to initiate the replication and release of new viral particles. The release of damage-associated molecular patterns (DAMP) along with microorganism-associated molecular patterns (MAMPs) is followed by the host’s pattern-recognition receptors (PRRs) recognizing the neighboring airway cells, and the recruitment of a multitude of immune cells, including APCs, which present SARS-CoV-2 antigens during the generation of adaptive immune responses. Hence, the initial immune response characterized by the activation of innate immunity is followed by a virus-specific adaptive immune response.

**Figure 1:**
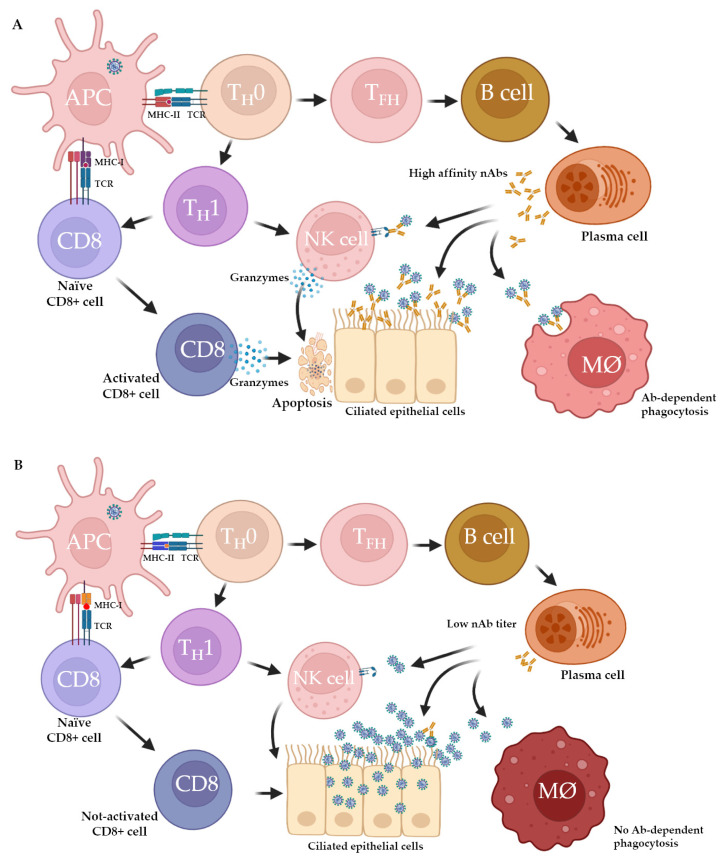
HLA variation determines SARS-CoV-2-specific antibody response. In this example, we present different scenarios suggesting differential SARS-CoV-2-specific antibody response impacted by variation in the HLA class II molecule. (**A**) MHC-II molecule binding to SARS-CoV-2 peptides induces efficient CD4+ T cell and antibody response leading to viral neutralization, ADCC, and the death of SARS-CoV-2 infected cells. (**B**) Polymorphic MHC-II molecule binding to viral peptide fails to stimulate CD4+ T cells and results in low antibody titer, lack of ADCC, and ADP. Abbreviations: Ab: antibody; ADCC: antibody-dependent cellular (NK cell) cytotoxicity; ADP: antibody-dependent phagocytosis; HLA: human leukocyte antigen; MØ: macrophage; MHC: major histocompatibility complex; nAbs: neutralizing antibodies; T_H_0: CD4+ helper cells; T_H_: T helper; T_FH_: T follicular helper cells. Figure made using BioRender.

During the generation of adaptive immune responses, the HLA molecules are involved in SARS-CoV-2 antigen presentation [32,33]. Following SARS-CoV-2 infection or vaccination, the spike antigen is taken up by antigen presenting cells, such as dendritic cells, macrophages, and B-cells, and processed into smaller peptides. As depicted in Figure 1, dendritic cells and alveolar macrophages present the peptides associated with HLA class I molecules to the T cell receptor (TCR) of cytotoxic CD8+ T cells (CTLs), leading to the death of SARS-CoV-2 infected cells [34,35,36]. On the other hand, dendritic cells present the peptides with HLA class II molecules to the TCR of naïve CD4+ helper (T_H_0) cells that differentiate into T follicular helper cells (T_FH_) that, in turn, induce B cell differentiation in the germinal center of draining lymph nodes that will eventually mature into memory B cells and plasma cells [37,38]. The plasma cells secrete SARS-CoV-2 specific neutralizing antibodies that block the interaction between the virus and the ACE-2 receptor [39,40]. In addition to inducing B cell differentiation, CD4+ T cells also induce the activation of CD8+ CTLs [41,42]. Differences in antibody responses have been observed based on the antigen target, including the spike protein and its RBD, and the nucleocapsid protein (NP), with the former correlating with viral neutralization [43,44]. Additionally, antibodies may play a protective role through the mechanisms involving antibody-mediated phagocytosis (AMP) or antibody-dependent cellular cytotoxicity (ADCC), by involving macrophages and natural killer cells, respectively [43,44]. Although immunoglobulin G (IgG) are relevant to durable humoral immune responses, immunoglobulin M (IgM) isotypes play a significant role during the acute phase of infection, and immunoglobulin A (IgA) antibodies are involved in the mucosal defense against SARS-CoV-2 [43,44]. The activation of SARS-CoV-2-specific immune cells leads to the death of infected cells, and the majority of patients subsequently clear the virus and recover [36]. In contrast, in those who develop severe disease, cytokines and chemokines continue to attract monocytes, macrophages, neutrophils, and T cells to the site of the infection, promoting further inflammation and the uncontrolled production of pro-inflammatory cytokines (also known as a cytokine storm), vascular endotheliitis, thrombosis, and angiogenesis [31,45,46].

One of the most peculiar characteristics of SARS-CoV-2 infection, in terms of the humoral antibody responses, is the extreme inter-individual variability. Although the exact underlying mechanism(s) have not been fully elucidated, the observed inter-individual variability in antibody responses has previously been associated with age [47,48], sex [48], comorbid conditions [48,49,50,51], smoking [47,48], COVID-19 severity [51,52], medications [49,50], viral lineage [53,54], SARS-CoV-2 vaccination type and dose [28,55,56], hybrid immunity [57,58], and time since SARS-CoV-2 infection or vaccination [59]. HLA alleles exhibit a high degree of variation [9]. Whether the differences observed in SARS-CoV-2 specific antibody responses between individuals are due to differences in the capability of the HLA molecules in presenting several peptides is not fully understood.

## 3. HLA Variation and Humoral Immunity in SARS-CoV-2

We conducted a literature search of the databases in PubMed and Google Scholar with the keywords “HLA” and “antibody response”, or “immunoglobulin response”, in individuals following “SARS-CoV-2 infection”, or “COVID-19”, and/or “vaccination”. The study cohort characteristics, including infection status, vaccination status, number of study participants and date, and antibody response-type determinations are summarized in Table 1. There was extreme heterogeneity in the study designs, sample sizes, and duration of follow-up [60,61,62,63,64,65,66,67,68,69]. In addition, the humoral immune responses were assessed by measuring antibody titers based on anti-RBD IgG isotypes in two-thirds of the studies [60,61,62,64,66,68], and three studies determined anti-Spike IgG isotype levels [61,63,65]. In another study, the antibody levels evaluated included anti-RBD IgA isotype and anti-NP total Ig levels [66]. Only three studies determined the levels of neutralizing antibodies [64,66,67]. Whereas high antibody responders were defined as those having the top 25th or 33rd percentile of the titer distribution [60,64,67], low antibody response was defined as having the lowest 5th or 33rd percentile of the titer distribution [65,68]. Other investigators compared differences in the median or mean titers between carriers and non-carriers [61,62,63,64,65,66,67]. While all the studies included MHC class II association with antibody responses, only five studied MHC class I association with antibody responses. Except for one study [61] which included children, all the remaining studies included adults only.

**Figure 2:**
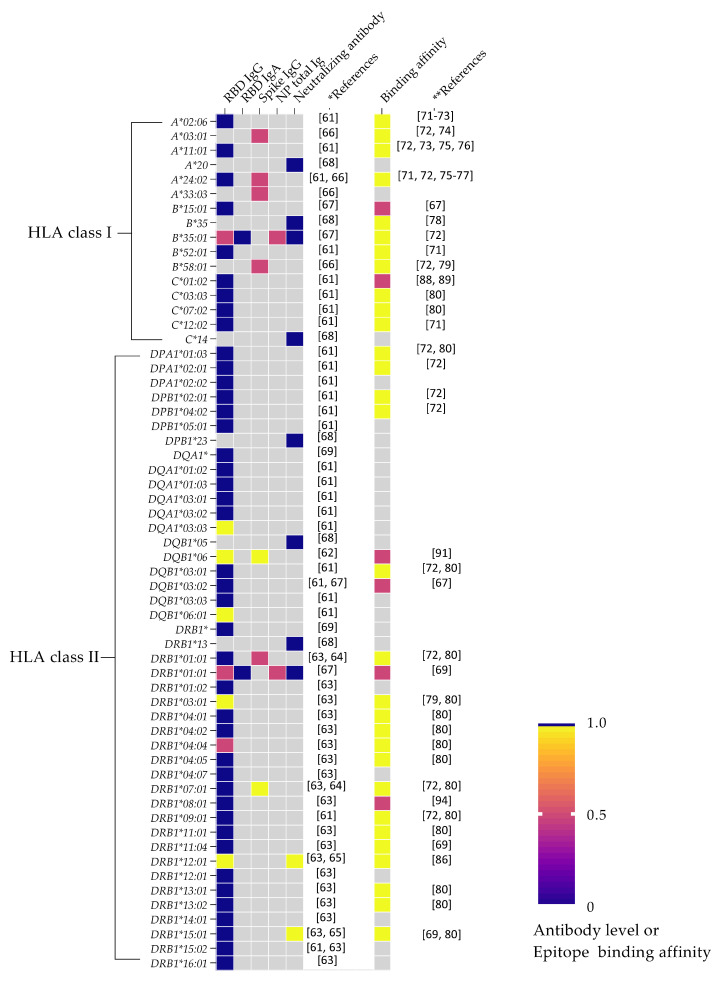
Heat-map showing the association between HLA variants and antibody responses or epitope binding affinity. Associations are depicted arbitrarily as high (scaled 1, yellow boxes), low (scaled 0.5, red boxes), or no association (scaled 0, dark-blue boxes). Gray boxes indicate undetermined values. References shown are for antibody response* or epitope binding affinity** studies.

Though research on the association between HLA variants and humoral immune responses is emerging, the reports revealed conflicting results (Figure 2). Several recent studies provided evidence that specific HLA variation, namely DQA1*03:03, DQB1*06, DRB1*03:01, or DRB1*07:01, enhanced the serological response post vaccination [60,61,62,63,64]. In a study conducted in Japan involving 100 health care workers, vaccination with the BNT162b2 vaccine was followed by a significant anti-RBD IgG response in individuals with the DQA1*03:03:01 haplotype [60]. Interestingly, individuals who received two doses of the BNT162b2 vaccine and carry the DQB1*06:01 allele also showed protection against the decline of anti-RBD IgG titers [60]. A recent study by Mentzer and colleagues in the UK performed one of the most detailed HLA class II genomic analyses with the largest sample size (*n* = 2753) undertaken to date [61]. In this study, the investigators reported significantly higher levels of anti-RBD and/or anti-S IgG antibody responses in individuals carrying.

DQB1*06 alleles who received the ChAdOx1 one vaccine dose or were boosted with the same vaccine, or the BNT162b2 or mRNA-1273 vaccines. Interestingly, individuals carrying the DQB1*06 allele were less likely to exhibit breakthrough infections compared to non-carriers. Furthermore, memory B-cell responses in DQB1*06 carriers were increased following vaccination, and anti-RBD IgG production in a cohort of individuals who received a booster vaccination persisted for several months. In another study of 420 UK participants who received a single dose of the BNT162b2 vaccine post natural infection, individuals with the DRB1*03:01 allele demonstrated higher titers of anti-RBD IgG, although the antibody titer abated (average of 121 days) after a second dose of the same vaccine, despite sustained T cell responses against the spike protein [62]. Interestingly, individuals carrying the DRB1*13:02 allele in this study exhibited a greater susceptibility to symptomatic disease [62]. Similarly, a study conducted in Spain demonstrated high anti-Spike IgG titers among 87 individuals with the DRB1*07:01 allele and DRB1*07:01~DQA1*02:01~DQB1*02:02 haplotype 30 days after a second dose of the mRNA-1273 SARS-CoV-2 vaccine [63]. A recent study by Higuchi et al. of 87 Japanese patients with rheumatoid arthritis, who were vaccinated with the BNT162b2 vaccine, demonstrated that DRB1*12:01 allele carrier frequency was higher in those individuals with high anti-RBD and neutralizing antibody responders [64]. Additionally, allele carrier frequencies of DRB1*15:01 were higher in those individuals with high neutralizing antibody responses [64].

Other studies have documented the HLA variations that led to a reduced serological response post vaccination or post infection. Cocchiolo et al. studied 111 individuals without prior infection who received two BNT162b2 SARS-CoV-2 vaccine doses [65]. This study documented a weak anti-Spike IgG response that was less than 5% of the lowest community antibody response 14+ days after the second vaccine dose, in individuals carrying A*03:01, A*33:03, and B*58:01 alleles, and A*24:02~C*07:01~B*18:01~DRB1*11:04 haplotype. Another study by Fischer et al. investigated 119 COVID-19 convalescent adults, with a median follow-up of 250 days, who were unvaccinated [66]. In this study, individuals with the the B*35:01 and DRB1*01:01 alleles exhibited reduced titers of anti-RBD IgG and anti-NP total IgG. Likewise, Gutiérrez-Bautista et al. demonstrated that individuals carrying the allele DRB1*01:01 exhibited low anti-S antibody levels 30 days after a second dose of the mRNA-1273 vaccine [63]. Despite lower antibody levels, both alleles were also associated with a shorter duration of COVID-19 disease, suggesting the protective role of specific HLA variations. Notably, it is assumed that higher antibody levels are generally associated with protection. However, previous studies have documented that higher antibody titers can be associated with COVID-19 severity [43,44], although another study found that more severe illness was associated with higher ratios of anti-NP antibodies compared to anti-Spike/RBD antibodies [52]. Hence, whether the protective effects of B*35:01 and DRB1*01:01 alleles on COVID-19 clinical outcomes [66] is related to the generation of antibodies remains to be determined. Further, a low anti-Spike IgG titer was noted in the UK study in individuals carrying the DRB1*04:04 variant after a single BNT162b2 mRNA-based vaccination in patients with prior SARS-CoV-2 infection [62].

Contrary to the evidence presented above, there is literature that supports the lack of an association between antibody response and HLA variation. In the study conducted in Japan by Khor et al., no association was shown between anti-RBD IgG responses and polymorphisms in the MHC class I HLA locus-A*, -B*, -C*, DPA1*, DPB1*, DQA1*, DQB1*, and DRB1* [60]. Likewise, Astbury et al. showed no association between antibody production and several DRB1* alleles [62]. Notably, this study demonstrated no association between anti-RBD response and the DRB1*07:01 allele, although in a previous study, DRB1*07:01 was associated with high anti-S antibody responses [63]. Fisher et al. also demonstrated that there was no association between B*15:01 or DQB1*03:02 and anti-RBD responses, nor between B*35:01 or DRB1*01:01 and anti-RBD IgA and neutralizing antibodies after natural SARS-CoV-2 infection [66]. Another study undertaken in Austria found no association between neutralizing IgG antibodies and COVID-19 patients (*n* = 84) with A*20, B*35, C*14, DPB1*23, DQB1*05, and DRB1*13 polymorphisms [67]. A smaller study involving 56 patients conducted in Italy by Ragone et al. demonstrated no association between DRB1* or DQA1* and anti-RBD IgG titers in healthy individuals, measured two weeks to four months after two doses of BNT162b2 vaccination [68]. Another recent study conducted in Germany, in albeit a small number of COVID-19 patients (*n* = 49) who were either unvaccinated or received the BNT162b2 or ChAdOx1 vaccines, demonstrated that there was no association between A*01, A*02, A*24, B*44, C*04, C*07, DP*04, DQ*03, DQ*05, DQ*06, or DR*13 and anti-RBD IgG responses [69].

## 4. SARS-CoV-2 Peptide Epitope Binding Affinity and Antibody Response

HLA molecules can only present SARS-CoV-2 peptides that have binding epitopes compatible with its specific antigen binding cleft [29,30,31]. This leads to a mechanism whereby different HLA alleles present diverse peptides derived from the same SARS-CoV-2 antigen. Having certain alleles can make the SARS-CoV-2 antigenic presentation more efficient, leading to a better humoral immune response, both in terms of quantity and breadth (or affinity). Thus, the presence of HLA molecules capable of presenting several peptides will lead to a more efficient humoral immune response with a higher antibody titer. Antibodies that functionally neutralize correlate with protective capacity [36,43,44]. On the contrary, some unique HLA variants with the capacity to handle a very limited number of antigens are faced with the consequences of an inefficient humoral immune response. Thus, the differences in the capabilities of the HLA alleles in handling SARS-CoV-2 antigens may be the reason for the observed differences in anti-SARS-CoV-2-specific immune responses between individuals, the clinical severity of COVID-19, and vaccine efficacy.

The peptide-binding affinity of HLA class I alleles shows diverse characteristics. Notably, none of the HLA class I variants were associated with a positive SARS-CoV-2-specific antibody generation [60,65,66,67]. For example, A*02:06, A*03:01, A*11:01, A*24:02, B*35, B*35:01, B*52:01, B*58:01, C*03:03, C*07:02, and C*12:02 are associated with low or no antibody production, despite binding strongly with their respective peptide fragments (Figure 2) [70,71,72,73,74,75,76,77,78,79]. Additionally, A*02:06, B*35:01, and B*58:01 have been shown to be protective against SARS-CoV-2 infection [66,72,78]. On the contrary, A*03:01, A*11:01, A*24:02, B*52:01, and C*12:02 are all associated with an increased risk for severe COVID-19 and to susceptibility for infection with SARS-CoV-2 [72,73,76,80,81,82,83,84,85,86]. B*15:01 and C*01:02 are unable to present sufficient amounts of peptide epitope [66,87,88], and these alleles are also associated with severe COVID-19, COVID-19-related death, or an increased susceptibility for SARS-CoV-2 infection [66,75,88,89]. Whether the association of these HLA class I variants with adverse COVID-19 outcomes is attributed to their lack of antibody and/or efficient CD8+ CTL response deserves further study. Hence, the most plausible explanation for MHC class I roles as a protective or detrimental allele might be related to their ability to modulate the immune response in an antibody-independent manner.

With regards to the MHC class II, peptide-binding prediction analyses have revealed that DRB1*03:01, DRB1*07:01, and DRB1*12:01 are all associated with a significant increase in SARS-CoV-2-specific antibody responses following the administration of mRNA SARS-CoV-2 vaccines [62,63,64], and are also able to bind epitope peptides with a strong affinity [68,71,78,79]. Interestingly, these alleles are associated with protection against severe COVID-19 as well as reduced susceptibility to SARS-CoV-2 infection [66,72,78,81,85,89,90]. Hence, the protection against and/or reduced susceptibility to COVID-19 in individuals carrying these alleles could be attributed, at least in part, to the strong peptide binding affinity and the increase in antibody responses. Although several HLA class II variants, including DPA1*01:03, DPA1*02:01, DPB1*02:01, DPB1*04:02, DQB1*03:01, DRB1*01:01, DRB1*04:01, DRB1*04:02, DRB1*04:05, DRB1*09:01, DRB1*11:01, DRB1*11:04, DRB1*13:01, DRB1*13:02, and DRB1*15:01, are able to bind SARS-CoV-2 peptides with a strong affinity [71,79,85], there is no association between these alleles and antibody production [60,62]. On the contrary, the study by Higuchi et al. demonstrated that DRB1*15:01 is associated with increased anti-RBD neutralizing antibody responses [64], and exhibits a strong epitope binding affinity [68,79]. However, it is not associated with protection against severe COVID-19 [89]. DRB1*04:01 and DRB1*11:04 are both protective against severe COVID-19 and associated with a reduced susceptibility risk against SARS-CoV-2 infection [85,90,91]. Hence, the protective role of these alleles might be mediated by antibody-independent mechanisms, such as through CD4+ and/or CD8+ immune responses. DRB1*09:01, DRB1*11:01, DRB1*13:01, DRB1*13:02, and DRB1*15:01 are all associated with an increased risk of severe COVID-19 [62,89,90,92], and may also operate through alternative pathways. Hence, the effect of the above HLA variants might be attributed to their impact on T cell responses rather than on humoral immunity. This notion is supported by the study conducted by Astbury et al., who demonstrated that DRB1*15:01 carriers exhibited a significant increase in T-cell response, assessed using an IFN-Ɣ ELISpot assay, despite the absence of humoral immunity [62]. DRB1*01:01 and DRB1*04:04 exhibit a strong peptide binding affinity [71,79]. However, both these alleles are associated with a low or no association in antibody production [62,66]. In particular, DRB1*01:01 is associated with a shorter disease duration and protection against severe COVID-19 [66]. Hence, its protective effects might be related to its actions through antibody-independent mechanistic pathways involving CD8+ CTLs. Whereas one report revealed no association between DRB1*07:01 and the anti-RBD level [62], another study demonstrated that it is associated with increased anti-S levels [63], which might be attributed to its ability to bind peptide epitopes strongly [71,79]. In addition, DRB1*07:01 is associated with protection against severe COVID-19 as well as reduced susceptibility risk for SARS-CoV-2 infection [89,90].

Finally, DQB1*06 is associated with a significant increase in anti-RBD and/or Spike IgG responses in ChAdOx1 or BNT162b2 vaccinated individuals [61], and exhibits low peptide-binding affinity [90]. Although Mentzer et al. demonstrated the reduced hazard of breakthrough infection in vaccinated individuals carrying DQB1*06, this allele has been associated with an increased susceptibility for SARS-CoV-2 infection in a previous study [90]. As noted in an earlier section of this review (Figure 2), several HLA class II variants have been associated with low or weak antibody responses [60,62,66]. A SARS-CoV-2 peptide-binding prediction analysis demonstrated that DQB1*03:02, DRB1*01:01, and DRB1*08:01 alleles are unable to bind viral peptides with high affinity [65,67,91], have a weak or no antibody response [62,66], and are associated with an increased risk for severe COVID-19 and susceptibility for infection with SARS-CoV-2 [66,71,78,81,91,93]. Taken together, the association of DQB1*03:02, DRB1*01:01, and DRB1*08:01 alleles with low SARS-CoV-2 epitope-binding affinity may lead to low or no antibody production, and increase the risk of individuals for severe COVID-19 outcomes. Additionally, *DRB1:01:01* can induce regulatory T cell responses that may inhibit downstream humoral immune response and antibody generation [94].

## 5. Conclusions and Future Prospects

The studies reviewed here reveal the inter-individual variations in antibody responses associated with diverse HLA polymorphisms [60,61,62,63,64,65,66,67,68,69]. There are several study design factors that lead to variable results within the literature, including differences in study cohort characteristics, the timeframe in which antibody levels were assessed, different instrumentation and cutoffs used for antibody assessment, and types of antibodies assessed (isotypes, antigen targets, and neutralizing ability) (Table 1). Small sample sizes and extreme allelic heterogeneity pose significant power limitations. The lack of standardization within SARS-CoV-2 antibody assays limits comparisons between studies. Further, differences in vaccine types, SARS-CoV-2 variants, genotyping methods, and algorithms for predicting HLA haplotype may also impact results [95,96]. For instance, a range of sequencing or genotyping technologies were used, including Ion GeneStudio [97], PacBio [98], Illumina, and Affymetrix [99,100,101]. The targeted next-generation sequencing panels and/or differences in the PCR techniques applied had different levels of resolution for HLA calling. An array of different algorithms were used to predict the HLA genotype, including NGSengine [99], AllType NGS [102], TypeStream Visual [103], Imputation, and PHASE [104,105,106], each with different technical performance parameters which also limit comparisons between studies.

Given that the CD4+ T_FH_ TCR:MHC class II interaction is necessary for the generation of antibodies, any associations found between MHC class I and antibody titers [45,46] may imply a coincidental rather than a causal association. This notion is supported by the fact that in all the studies included in this review, HLA class I polymorphisms were not observed to have any effect on SARS-CoV-2-specific antibody responses [61,66,67,68]. On the other hand, in the event that future studies demonstrate an association between HLA class I and antibody responses, the findings might be the result of indirect regulatory pathways rather than a direct CD8 + TCR:MHC class I interaction. For instance, this notion is supported by the observation that *HLA-B* encodes a microRNA that regulates IgA production [107]. Indeed, the fact that some HLA class I alleles have no effect on antibody generation despite a strong peptide binding affinity implies that such alleles modulate the immune response in an antibody-independent manner through effects on CD8+ CTL responses.

Notably, two-thirds of the studies documented associations between HLA class II polymorphisms and antibody responses [60,61,62,63,64,65,66]. In addition, alternative immune-genetic pathways may be involved in the generation of antibodies. Indeed, a more recent report demonstrated that genetic variation in the 3′ regulatory region 1 (3′RR1) of the human immunoglobulin heavy chain locus has been associated with significant effects on SARS-CoV-2-specific antibody responses following BNT162b2 mRNA vaccination [108]. Whereas single nucleotide polymorphisms (SNPs) in rs373084296, rs7494441, rs12896746, rs12896897, and rs7144089 of the *3′RR1* of the human immunoglobulin heavy chain were associated with high levels of antibodies, SNPs in rs12896746, rs12896897, and rs7144089 were linked to low levels of antibodies (<10th percentile). Alternatively, immunogenicity demonstrated in response to SARS-CoV-2 may be ascribed to the generation of T-cell responses without sufficient humoral immune responses [62], or more probably due to the indirect pathways and impact by non-MHC-related regulatory genes [107,109]. Indeed, a more recent study demonstrated that non-MHC genes were associated with significant antibody production in Italian health care workers who received a second dose of the BNT162b2 mRNA-based or ChAdOx1 adenovirus-based SARS-CoV-2 vaccination [110]. The gene variants involved included *TP53* (rs1042522), *ABO* (rs657152), *APOE* (rs7412/rs429358), *ACE2* (rs2285666), *HLA-A* (rs2571381/rs2499), and *CRP* (rs2808635/rs876538). All these alleles were associated with significant increases in anti-spike IgG, as well as neutralizing antibodies, between two weeks and six months post vaccination.

In addition, recent genome-wide association studies (GWAS) have identified several SNPs, including the *BCL11A* (rs1123573) and *TAC4* (rs77534576) genes, that are associated with COVID-19 severity [111]. These genes are involved in B-cell lymphopoiesis, and the role variants in these loci play in SARS-CoV-2-specific antibody responses deserves further investigation. Other variations or SNPs related to the host’s PRRs could also affect antibody responses. For example, PRRs such as the *Toll-like receptor 7* (*TLR7*) are essential for immune cell activation and the connection to antimicrobial adaptive immunity [112,113]. Hence, individuals with unique loss-of-function (LoF) variants in *TLR7* (rs189681811 and rs147244662) that are associated with COVID-19 severity have been linked with an abrogated production of IFNI and II [112]. Whether such early antiviral immune responses related to LoF variants in *TLR7* also affect subsequent SARS-CoV-2-specific humoral immunity remains to be elucidated.

In addition, several authorities have demonstrated the emergence of SARS-CoV-2 variants with an enhanced capability to circumvent antibody responses [114,115,116,117,118]. These emerging SARS-CoV-2 variants may also have the potential to interact with HLA molecules with different binding affinity characteristics. Indeed, recent studies have revealed a dramatic loss of peptide-binding affinity associated with a mutation of the spike protein in individuals carrying A*02:01, B*07:02, DRB1*03:01, and DRB1*15:01 alleles [58,119,120,121,122]. Taken together, the emergence of new SARS-CoV-2 variants will pose a significant challenge to the development of effective immune responses and warrants further investigation.

In conclusion, the HLA loci are highly heterogeneous [9], and differences in study design, methodological approaches, and biological complexity have made this region difficult to study [95,96]. Thus, further studies using larger cohorts are needed to determine if there are associations between HLA loci and the SARS-CoV-2-specific immune response. It is expected that GWAS will provide further evidence about how genetic variation influences the differential antibody response after SARS-CoV-2 infection and/or vaccination. However, the molecular pathways involved remain to be elucidated. Replication studies intended to investigate the functional interactions of causal variants are underway by our group [123]. Ultimately, identifying the underlying immune-genetic mechanisms will pave the way for the optimization of new diagnostic modalities and the development of improved vaccines and therapeutic options against SARS-CoV-2 and other infectious diseases.

## Figures and Tables

**Table 1:** Studies on HLA variants and association with antibody responses.

Study Cohort Characteristics			Antibody Response		References
Infection Status	Vaccination	Number of Individuals	Date Antibody Response Assessed	Antibody Response Assessed	
No prior infection	BNT162b2	100	7 and 39 days after second vaccine dose	Anti-RBD IgG	[60]
With or without prior infection	ChAdOx1 or ChAdOx1+ BNT162b2 or mRNA-1273 or NVX-CoV2373	1076 or 1677	28 days after first vaccine dose or 28 to 184 days after second vaccine dose	Anti-RBD and/or anti-S IgG	[61]
With or without prior infection	BNT162b2	420	Unknown	Anti-RBD IgG	[62]
No prior infection	mRNA1273	87	30 days after second vaccine dose	Anti-S IgG	[63]
No prior infection	BNT162b2	87	144 days after second vaccine dose	Anti-RBD IgG and Neutralizing Antibodies	[64]
No prior infection	BNT162b2	111	14+ days after second vaccine dose	Anti-S IgG	[65]
Prior infection	None	119	>46 to >97 days after end of symptoms	Anti-RBD IgG, Anti-RBD IgA, Anti-NP total Ig, and Neutralizing antibodies	[66]
Prior Infection	None	84	26 and 61 days after symptom onset	Neutralizing antibodies	[67]
No prior infection	BNT162b2	56	2 weeks to 4 months after second vaccine dose	Anti-RBD IgG	[68]
Prior infection	With or without BNT162b2 or ChAdOx1	49	12 months after PCR positivity	Anti-RBD IgG	[69]

Abbreviations: Ig: immunoglobulin; NP: nucleocapsid protein; RBD: receptor-binding domain; S: spike antigen.

## Data Availability

Not applicable.

## Abbreviations

Antibody-dependent cellular cytotoxicity (ADCC); antibody-mediated phagocytosis (AMP); antigen-presenting cells (APCs); binding antibody unit (BAU); Coronavirus disease 2019 (COVID-19); cytotoxic T lymphocytes (CTLs); genome-wide association studies (GWAS); damage-associated molecular patterns (DAMP); human leukocyte antigen (HLA); loss-of-function (LoF); major histocompatibility complex (MHC); microorganism-associated molecular patterns (MAMPs); nucleocapsid protein (NP); pattern-recognition receptors (PRRs); severe acute respiratory syndrome coronavirus-2 (SARS-CoV-2); receptor-binding domain (RBD); single nucleotide polymorphisms (SNPs); spike antigen (S); T cell receptor (TCR); T follicular helper cells (T_FH_); regulatory T cells (Treg).
